# The Impact of COVID 19 on University Staff and Students from Iberoamerica: Online Learning and Teaching Experience

**DOI:** 10.3390/ijerph18115820

**Published:** 2021-05-28

**Authors:** Mario Jojoa, Esther Lazaro, Begonya Garcia-Zapirain, Marino J. Gonzalez, Elena Urizar

**Affiliations:** 1 Engineering Faculty, Department of Computer Science, Electronics and Telecommunications University of Deusto, 48014 Bilbao, Spain; mariojojoa@deusto.es (M.J.); mbgarciazapi@deusto.es (B.G.-Z.); 2 Faculty of Health Sciences, Valencian International University, 46002 Valencia, Spain; 3 Unit of Public Policy, Simon Bolivar University, Caracas 89000, Venezuela; margonza@usb.ve; 4 Deusto Business School Health, University of Deusto, 48014 Bilbao, Spain; elena.urizar@deusto.es

**Keywords:** COVID-19, mental health, university students, university staff, online learning

## Abstract

(1) Background: The COVID-19 pandemic has created a great impact on mental health in society. Considering the little attention paid by scientific studies to either students or university staff during lockdown, the current study has two aims: (a) to analyze the evolution of mental health and (b) to identify predictors of educational/professional experience and online learning/teaching experience. (2) Methods: 1084 university students and 554 staff in total from four different countries (Spain, Colombia, Chile and Nicaragua) participated in the study, affiliated with nine different universities, four of them Spanish and one of which was online. We used an online survey known as LockedDown, which consists of 82 items, analyzed with classical multiple regression analyses and machine learning techniques. (3) Results: Stress level and feelings of anxiety and depression of students and staff either increased or remained over the weeks. A better online learning experience for university students was associated with the age, perception of the experience as beneficial and support of the university. (4) Conclusions: The study has shown evidence of the emotional impact and quality of life for both students and staff. For students, the evolution of feelings of anxiety and depression, as well as the support offered by the university affected the educational experience and online learning. For staff who experienced a positive professional experience, with access to services and products, the quality-of-life levels were maintained.

## 1. Introduction

In early 2020, Severe Acute Respiratory Syndrome Coronavirus 2 (SARS-CoV-2) shocked the world, causing a worldwide pandemic, and on 11 March 2020, the World Health Organization (WHO) declared a global public health emergency [1]. The new coronavirus apparently started to spread in China during December 2019, before moving to different countries such as Thailand, Japan, the Republic of Korea (first confirmed cases on 20 January 2020), then to the United States, Vietnam, Singapore and, in late January 2020, to Australia, Nepal, Europe (first cases in France on 25 January 2020 and later in Germany, Finland, Italy, etc.), Malaysia, Canada, the Middle East, and other countries of the Western Pacific Region and South-East Asia, and onwards to Russia, Africa, and Latin America [1]. 

Soon after the outbreak of COVID-19 began to spread, many countries around the globe implemented control and social distancing measures. The main features of these control measures have been analyzed by the University of Oxford [2], according to their Coronavirus Government Response Tracker, and reflected in the Government Response Stringency Index (GRSI). This is a composite measure based on nine response indicators, including school closures, workplace closures, and travel bans, rescaled to a value from 0 to 100 (100 = strictest). If policies vary at the subnational level, the index is shown as the response level of the strictest sub-region.

Control measures for the first wave of the COVID-19 pandemic in Spain can be divided into two stages: Phase (1)—the pre-lockdown period, extending from January 31 (day of the first measure) to March 13. The first measure consisted of the start of a coordinated government public information campaign, using traditional media and social networks. On March 9, the closure of all educational institutions was established, as well as the closure of workplaces, and the start of teleworking. With all these measures, the GRSI rose to 25. On March 10, the cancellation of public events was approved, as well as the prohibition of meetings attended by over 1000 people and the arrival of international travelers from selected countries. This brought the GRSI to 45. Phase (2)—the lockdown phase, which started on March 14, when the state of alert came into effect throughout Spain. As of this day, people were only allowed to leave their homes for essential activities. The closing of businesses in certain productive sectors, and the recommendation to close public transport were also approved. With these measures, the GSRI rose to 68. In the case of one of the countries in Latin America, Colombia, the virus was confirmed to have reached it on 6 March 2020. On 12 March, four more cases were confirmed. Authorities declared a health emergency, suspending all public events involving more than 500 people. On the night of 15 March, the Ministry of Health and Education announced the suspension of classes for all state and private schools and universities in the country [2].

Quarantines have been employed throughout history to control the spread of infectious diseases such as cholera, the plague or, more recently, SARS and Ebola [3]. However, the current confinement of the whole population across countries is unprecedented [4]. It is also generally accepted that this lockdown can lead to substantial adverse consequences, including severe issues related to economic damage, legal and ethical issues, and also psychological effects on the population being confined.

As far as health is concerned, the novel coronavirus SARS-CoV-2 has affected all age groups, with the worst manifestations and highest death rates being found among older individuals and patients with comorbidities [5,6]. Moreover, the COVID-19 pandemic has created all manners of unanticipated unrest in society, the economy and mental health [7,8]. In a Chinese study, less than a third of the subjects evidenced moderate or severe levels of stress, depression and anxiety [9]. In a Spanish study conducted during the first days of the COVID-19 pandemic, levels of stress, depression and anxiety were lower compared to a previously cited study [4], although an increase in these levels was observed when lockdown started. In an Italian study, higher levels of stress, anxiety and depression during the COVID-19 confinement were observed in comparison to the Spanish study, with 18% to 32% of participants evidencing high or very high levels [10].

Considering the usual high incidence of emotional disorders in university students, it can be expected that the current situation may cause a notable impact on this population [11,12,13]. Most countries affected by the pandemic imposed drastic measures on educational establishments, and the academic community has been greatly affected. Although the overall impact on education and mental health of the university environment is still unknown, it is expected to be very considerable [14,15]. There are few studies that focus on the impact of SARS-CoV-2 on the university environment, not only among students, but also among university staff, one of the published works on the subject [7] stating that around 24.9% of university students have experienced anxiety due to the pandemic. 

Given the possibility of future pandemics, it is important to understand the impact of the pandemic from a comprehensive perspective. As such, the aim of this paper is to help explore the personal, educational or professional experience and the online education or teaching lived during lockdown by students and staff within the Latin American university environment.

The current study has two aims: (1) to analyze the evolution of quality of life, stress, depression and feelings of anxiety in students and staff of universities during lockdown; and (2) to identify the predictors of educational/professional experience and online learning/teaching experience.

## 2. Materials and Methods

### 2.1. Survey Description

LockedDown [16] is an action promoted by the London School of Economics and Political Science—Health Policy and Health Bites to evaluate how the measures taken to combat the COVID-19 pandemic have affected the educational process, opportunities for professionals and the well-being of personnel linked to the university environment.

An ad hoc global survey was developed especially aimed at university staff (staff and students) for this purpose, in order to assess the influence of COVID-19 on social, labor, academic, economic and psychosocial variables and leisure. The original version of the survey was developed in English with the collaboration of a multidisciplinary team. For this study, the translation into Spanish was carried out by native speakers in Spanish from both Spain and the participating LATAM countries.The survey consisted of 82 items, 48 dichotomous (Yes/No), 34 multiple-choice nominal and ordinal items and a free response item to give respondents the possibility to add comments in an open response format. The approximate response time for all questions was 7–10 min.

The following section lists the variables included in the LockedDown survey and that will be analyzed: sociodemographic variables (age, gender, academic level, place of residence, etc.); work impact: employment/unemployment/job search; impact on personal and family economy; impact on social life; impact on quality of life, anxiety, stress; access to essential products and other services; family caregiver role, dependents, children; physical exercise/leisure and free time; physical or psychological abuse; impact on the university environment (online teaching, online study and class, exams).

### 2.2. Student Sample Description 

As can be seen in Table 1, 1084 students formed part of the study, 65% of whom were male, 83% undergraduate and 51% from Spain. Of the total sample, 66% say that their “Educational experience was negatively impacted” and 55% express the fact that "Online learning is possible but in person it is better". 

### 2.3. Staff Sample Description

In total, 554 people belonging to the staff of different universities participated in the survey, 60% of whom were female, 91% were staff from face-to-face university classes and 68% from Spain (Table 2). Of them, 50% affirmed that online teaching is possible but in person it is better and that their professional experience was either negatively impacted (37%), not impacted (35%) or positively impacted (23%). 

### 2.4. Ethical Issues

Ethics approval was obtained from the Research Ethics Committee of The London School of Economics, with the ethical issues raised by the proposed research having been properly taken into account and adequate safeguards put in place.

### 2.5. Methods

In the following sections, the methods used for the analysis of the data collected are described in order to extract information that allows for decision-making and future planning in the event of lockdowns. Mainly, two approaches were used, the first based on classical descriptive statistics and the second on machine learning. 

#### 2.5.1. Classic Descriptive Statistics

The data analysis was performed using the SPSS version 26.0 statistical package [17] in order to carry out the following analyses:

The descriptive analysis is displayed as the mean and standard deviation for quantitative variables, and frequencies or percentages for qualitative variables.

##### Multiple Linear Regression

A multiple linear regression statistic was applied in order to predict the educational/professional experience and the online learning/teaching experience from several variable predictors (multiple regression). The regression method was Enter. 

To control for multicollinearity, a VIF calculation was performed and a Durbin–Watson test statistic applied for tests based on the assumption of independent errors. The missing values were replaced by mean values.

##### CHAID (Chi-Square Automatic Interaction Detector) 

The data mining technique was applied, consisting of a statistical and multidirectional tree algorithm that explores data and creates segments and profiles. This also allows automatic detection of interactions using the Chi-square method. At each step, CHAID chooses the predictor variable that maintains the strongest interaction with the dependent variable [18].

#### 2.5.2. Machine Learning 

Automatic learning techniques are currently being widely used for tasks related to attribute selection [18] based on their important features in terms of predicting a response variable selected, among other applications. Different machine learning techniques were applied in this work in order to find the attributes that most affect behavior of the response variables professional/educational experience and online learning/teaching experience, as well as the positive or negative trend of the support vector machine coefficient in predicting the response variable analyzed. 

##### Reduction in Dimensionality Based on Pearson’s Correlation

**Figure 1:**
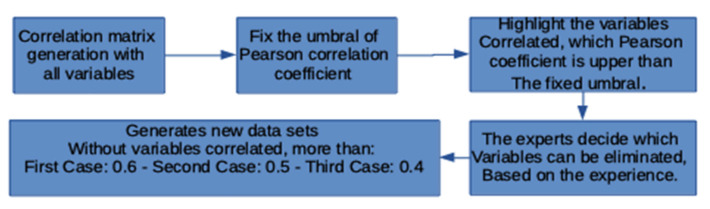
Block diagram of the steps to be taken to select correlated variables. Fix the threshold of Table 21. Logistic Regression [20] and Support Vector Machine [21].

According to [19], it is desirable in a machine learning analysis for input variables not to show any correlation, in order to reduce any disturbance or dispersion in the input matrix. In this respect, it proved necessary to obtain a Pearson correlation matrix for all fields so as to select those variables that exceeded the 0.4, 0.5 and 0.6 thresholds, respectively, and thus to define which of them should be preserved. A block diagram of the procedure undertaken is shown below (Figure 1). 

##### Random Forest as an Attribute Selector Algorithm 

This takes the form of a set of decision trees put together using the technique known as bagging, in order to increase the capacity for generalization and reduce variance in the performance metric required [22,23,24]. This constitutes one of the most commonly used metrics in the industry and is widely used to determine the importance of attributes. The functioning of this algorithm is mainly based on a calculation of the entropy of Equation (1) regarding the data for each tree, with a view to determining those variables that provide the most information in terms of the classification task.

$$Entropy=-P_{i}log_{2}\left(P_{i}\right)$$

**Figure 2:**
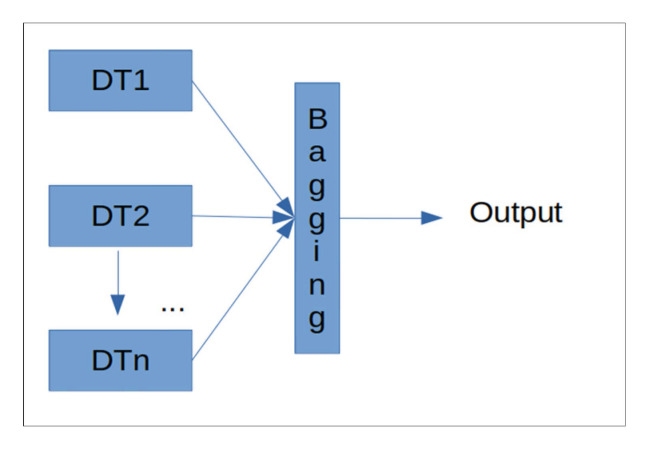
Attribute importance detection model based on decision trees used.

Thus, bagging proposes an algorithmic target to integrate machine learning algorithms in order to improve the general performance metrics of the system used. A block diagram is shown below of the model used (Figure 2). 

Each $DT_{n}$ block corresponds to a decision tree trained using an independent part of the data, the set of which is then put together in the last bagging block in inference time so as provide an agreed output. 

##### Multinomial Logistic Regression as an Attribute Selector Algorithm 

This is known as a regression technique used to predetermine a categorical variable—the reason why it fits perfectly into this work, as it attempts to predict the professional/educational and online learning/teaching variables, and to determine which attributes mainly interact in predicting them. Generally speaking, its functioning is based on data analysis according to a multinomial distribution, as shown below.

(1) $$f\left(x\right)=\frac{n!}{x1!\ldots \ldots xk!}p^{x1}\ldots \ldots p^{xk}$$

From this can be obtained a logarithmic probability ratio or Logits,

(2) $$P^{xi}=exp\left(\frac{\frac{Y_{i}}{N_{i}}}{X_{i}}\right)$$

which represents the rate of attributes of the $X_{i}\;$ array in the response variable $Y_{i}$ by making use of the Softmax function, shown in Equation (3) for this polychotomous case.

(3) $$Softmax\left(x\right)=\frac{e^{xi}}{\sum_{}^{}e^{xj}}$$

##### Support Vector Machine as an Attribute Selector Algorithm 

This constitutes one of the most commonly used algorithms in classification problems and categorical regression. Its simplicity and computational efficiency make it an ideal algorithm for these types of application that feature rapid deployment and great reliability. Its functioning is based on maximizing the margin (distance between the data support vectors used) in order to trace a hyperplane that represents the algorithm stage. In terms of inference, the relative position of the individual vectors is compared to the hyperplane and, hence, the extent to which it belongs to the different sets is defined. It is possible to access the model weights once the algorithm has been trained.

(4) $$H=W^{T}*X+B$$

where $W^{T}$ is a vector array whose direction focuses on the solution that is being sought. Therefore, the importance of the feature can be determined by comparing the size of these coefficients to each other. This, in turn, means that the main features used in the classification can be identified and those not considered important can be eliminated (i.e., those that have the least variance). 

Reducing the number of attributes in machine learning plays a very important role, especially when working with large data sets. Indeed, this may speed up the training, prevent overfitting and, ultimately, lead to better classification results thanks to the reduction in noise in the data.

## 3. Results

In this section, the data analysis will be carried out independently of classical descriptive statistics and machine learning. The results obtained using each of the methods presented for the variables are compared—namely, professional/educational experience and online learning/teaching—taking into account separately the group of students, on the one hand, and staff, on the other. A section showing the data analysis of the total sample is also included.

### 3.1. Student Results

#### 3.1.1. Evolution of Students’ Stress and Quality of Life 

**Figure 3:**
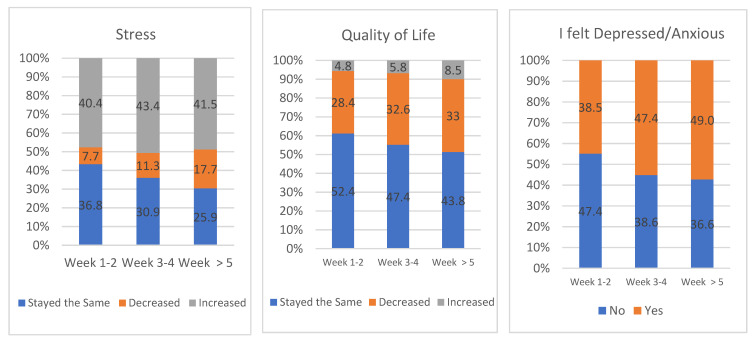
Evolution of stress, quality of life and feelings of depression and anxiety during lockdown (students).

Regarding the evolution of their stress level, quality of life and feelings of anxiety and depression, as reflected in Figure 3, it should be noted that most of the students experienced an increase in or maintaining of stress over the weeks, although the group in which less stress was reported also reported an increase throughout lockdown. Regarding quality of life, the majority perceive it to have been either maintained or decreased. Feelings of anxiety and/or depression increased as the weeks progressed.

#### 3.1.2. Educational Experience and Online Learning of Students: Multiple Linear Regression 

Table 3 shows the multiple linear regression results, exploring educational and online learning experience. For the educational experience variable, as in the case of the dependent one, the significant predictor variables were the following: age, social life, psychological or physical abuse, quality of life week 3–4, depression/anxiety week 1–2, teaching continuation, whether the university was supportive, whether the university progressed with exams, whether exams were postponed, financial difficulties, access to products and access to services. This model explains 16% of the variance.

In the case of the online learning experience variable, significant predictor variables were age, beneficial, teaching continuation, whether the university was supportive and whether exams were postponed. This model explains 20% of the variance.

### 3.2. Staff Results

#### 3.2.1. Evolution of Staff Stress and Quality of Life 

**Figure 4:**
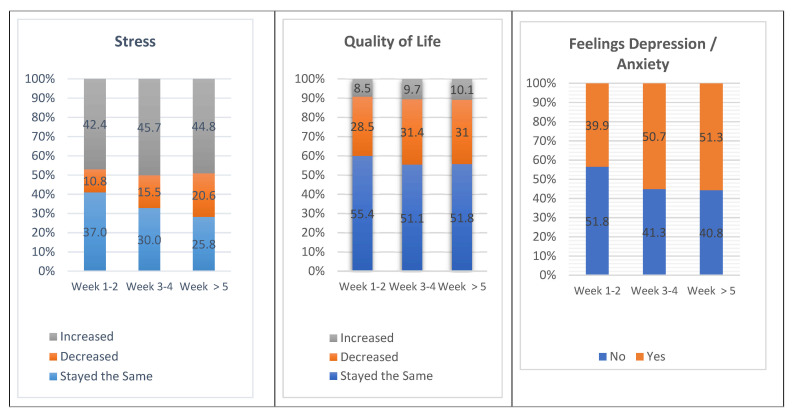
Evolution of stress, quality of life and feelings of depression and anxiety during lockdown (staff).

Most of the participants experienced an increase or the same level of stress as before lockdown. With regard to quality of life, half of the sample reported that it remained the same although, as in the case with the students, feelings of depression and anxiety increased throughout the weeks of lockdown (Figure 4).

#### 3.2.2. Multiple Linear Regression Staff Results

The multiple regression analysis reflected that of the professional experience dependent variable (Table 4), with significant predictor variables being as follows: country of residence, social life, stress week 1–2, depression/anxiety week >5 and beneficial. This model explains 16% of the variance. In the case of the online teaching experience dependent variable, the significant predictor variables were as follows: social life, stress week 1–2, teaching continuation, financial difficulties and loss of employment. This model explains 23% of the variance.

#### 3.2.3. Chi-Square Automatic Interaction Detector

**Figure 5:**
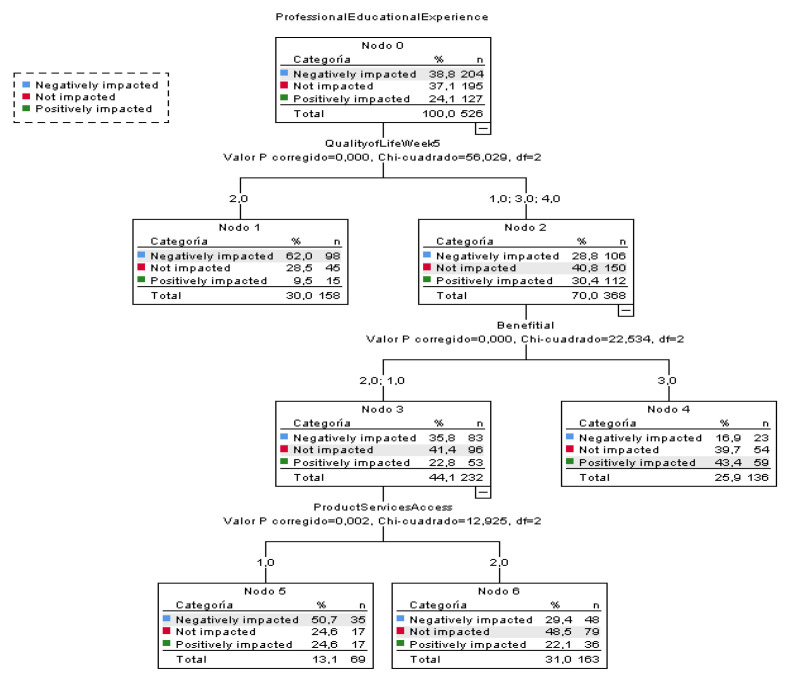
CHAID Analysis: dependent variable—professional experience of staff.

Figure 5 shows the results of the CHAID analysis in which the influence of different variables on the professional experience dependent variable is observed, according to classification of the participants into subgroups. The tree shows how the professional experience is either positive or the staff does not suffer any impact in the groups of participants with an increase in or maintaining of quality of life from week 5, the existence of benefits derived from lockdown, as well as access to services and products.

### 3.3. Machine Learning Results

Machine learning methods were applied in this section in order to analyze which variables were the most relevant among possible educational/professional experience and online teaching/learning variables. This was done separately both for the student subgroup and for the staff subgroup. The reduction in dimensionality via the elimination of correlated input attributes and the selection of relevant attributes for each output variable are described in the following sections.

Reduction in Dimensionality via Selection of Non-Correlated Attributes

It is recommended that any correlated information in input variables be eliminated in order to apply the machine learning algorithms in such a way as to leave no redundant information and in pursuit of optimum performance. The decision was thus made to use Pearson’s correlation coefficient as a metric, so as to ensure the reduction in dimensionality required. The results shown were obtained for the 0.6, 0.5 and 0.4 thresholds, respectively (Table 5).

Attribute Selection via Algorithm Committee

Three machine learning attribute selection models—Random Forest, Logistic Regression and Support Vector Machine—were run in order to select those attributes that most contribute to the response variables in question, in this case the educational/professional experience and online teaching/learning variables. Results were obtained from each intersect, with a view to ensuring an agreed response from the three algorithms. It is very important to highlight the fact that the data used were filtered according to the number of responses per country; in other words, only responses from countries that contributed with over 50 participants were recorded when dealing with the information gathering instrument. The results are shown below, classified according to the participant profile. 

#### 3.3.1. Student Results

The attributes selected by the algorithm committee are described in this section, using the student subset as input and the two proposed variables as output: educational experience and online learning experience.

Educational Experience of Students

The following table shows the attributes returned by the machine learning algorithm committee as output, taking the student dataset the input variable and the educational experience question as the output variable to be predicted (Table 6).

Learning Experience of Students

The following table shows the attributes returned by the machine learning algorithm committee as output, taking the student dataset as the input variable and the online learning question as the output variable to be predicted (Table 7). 

#### 3.3.2. Staff Results

The attributes selected by the algorithm committee are described in this section, using the staff subset as input and the two proposed variables as output: teaching experience and online professional experience.

Professional Experience of Staff

The following table shows the attributes returned by the machine learning algorithm committee as the output, taking the worker dataset as the input variable and the professional experience question as the output variable to be predicted (Table 8).

Online Teaching Experience of Staff

The following table shows the attributes returned by the machine learning algorithm committee as output, taking the worker dataset as the input variable and the online teaching question as the output variable to be predicted (Table 9). 

#### 3.3.3. Analysis for Profile of Student and Staff Set 

The attributes selected by the algorithm committee are described in this section, using the total dataset as the input and the two proposed variables as output: educational/professional experience and online learning/teaching.

Educational/Professional Experience of Students + Staff

The following table shows the attributes returned by the machine learning algorithm committee as the output, taking the worker and student dataset as the input variable and the educational/professional experience and online teaching questions as the output variable to be predicted (Table 10). 

As can be observed, of the set comprising 57 attributes, the committee draws attention to five as being the most relevant in predicting the educational/professional experience variable, with a positive trend existing in three of them and a negative trend in two of them.

Online Learning/Teaching Experience of Students + Staff

The following table shows the attributes returned by the machine learning algorithm committee as output, taking the worker and student dataset as the input variable and the online learning/teaching question as the output variable to be predicted (Table 11). 

As can be observed, of the set comprising 57 attributes, the committee draws attention to five as being the most relevant in predicting the educational/professional experience variable, with a positive trend existing in four of them and a negative trend in one of them. 

## 4. Discussion

The current study has two aims. On the one hand, the study analyzes the evolution of quality of life, stress, depression and feelings of anxiety in students and staff at universities in Latin America during lockdown. On the other hand, it aims to identify predictors of their educational or professional experience and online learning or teaching experience. 

### 4.1. Students

This study reveals that most students experienced an increase in or maintaining of stress, anxiety and depression over the weeks, while perceived quality of life decreased or was maintained. Another Spanish survey undertaken by 2530 members showed that moderate to extremely severe levels of anxiety, depression and stress were reported by 21%, 34% and 28% of respondents, respectively. In total, 50.43% of respondents evidenced a moderate to severe impact of the pandemic, especially students from Arts and Humanities and Social Sciences and Law [4]. The authors add that, in order to provide crisis-oriented psychological support and take preventive actions in other possible pandemics, mental health in university students should be carefully monitored. A further study carried out in Spain [24] found high levels of anxiety and depression (44.7 %) and severe anxiety and depression (31.6%). According to Cao et al. [7], around 24.9% of university students have experienced anxiety due to the pandemic. Living in urban areas, living with parents and having a stable family income appear to be protective factors for college students in combatting the anxiety experienced during the COVID-19 pandemic. However, having a family member infected with COVID-19 appears to be a risk factor in experiencing anxiety.

The study carried out on 381 university students in Jordan showed that most respondents experienced severe psychological distress (n = 265, 69.5%), while 209 students (54.9%) reported that they had no motivation for distance learning [25]. Among the predictors that were found to act as protective factors against higher levels of distress included older age and being strongly motivated to undertake distance learning. Another study found that more than half of students met the diagnostic criteria of generalized anxiety disorder (52%) and depression (63%) [26]. Similar results were found in other studies, which underline that safe coping strategies should be identified for students in order for them to face any pandemic-related challenges in the future and help ensure sustainable educational development in the world. Priority needs to be given to research regarding mental health, anxiety and students’ coping strategies along with psychological effects [27,28].

Of the total student sample analyzed in our study, 66% say that their “educational experience was negatively impacted” while 55% express the fact that "online learning is possible but in person it is better ". For its part, the study carried out among 133 students at the University of Dubai showed that 55% of the students liked distance learning and 26% would like to study 100% online, while the majority of students, 49%, were in favor of studying via a blended learning system, which is a combination of online and in-class teaching [26]. To this end, the authors support the view that the Ministry of Education should develop rules and guidelines to help universities continue to offer a blended learning system.

According to our multiple linear regression analysis, the educational experience was explained by the following variables: age, social life, psychological or physical abuse, quality of life week 3–4 and depression/anxiety week 1–2. In terms of university resources, the educational experience was associated with the continuity of teaching, and whether the university was supportive and whether they advanced with exams or they were postponed. Other predictor variables were financial difficulties and access to products and services. Machine learning analysis shows that educational experience was predicted by stress week 1–2 and >5, depression-anxiety week >5, quality of life week 3–4, financial difficulties, experience considered beneficial and whether the university was supportive and social life. Regarding the online learning experience, significant predictor variables according to the regression analysis were as follows: age, experience considered beneficial, continuity of teaching, whether the university was supportive and whether exams were postponed. According to the machine learning analysis, the variables that predicted online learning experience were as follows: stress week >5, experience considered beneficial, quality of life week 3–4 and whether the university was supportive.

An important finding of our study is that the evolution of feelings of anxiety and depression affected the educational experience and online learning, especially in the first week of lockdown and from week 5; that is, one month after lockdown. Although most students experienced an increase in or maintaining of stress, anxiety and depression over the weeks, the regression indicates that there is a greater educational experience when anxiety and depression levels are high in the first two weeks and then they are reduced from the first month after lockdown, reflecting an adaptive coping process with strong initial emotional impact and subsequent psychological adjustment. Other authors remark that coping strategies are directly associated with anxiety levels [27]. Students’ ability to seek social support, their isolation and mental disengagement and responsiveness to humanitarian issues were all assessed when measuring their copying strategies, while positive thinking, exercise and seeking support from family and friends were coping strategies that constituted predictors of less severe mental health impacts [27,28]. 

Social support is a basic protective factor, while lack of support from family, community and university social support was associated with depression, anxiety and stress of students [29]. Long periods of isolation and changes of routines are likely to provoke psychological and emotional distress, whereby perceived available peer support was negatively associated with depressive symptoms. Furthermore, positive and negative effects may mediate the association between students’ perceived available peer support and depressive symptoms during the pandemic [30].

The sudden adaptation to the online education model has been a challenge for many university students. Our study revealed that the educational experience was explained by the perception of the university as being supportive. In terms of the association between online learning experience and university resources and support, Islam et al. [31] state that the prevalence of mental health problems may be particularly high because of uncertainty in exams, classes, reopening of the university and strict social isolation. Along the same lines, Fawaz and Samaha [32] found a significant relationship between students’ satisfaction with online learning and the prevalence of depression, anxiety and stress symptoms, where satisfaction was also found to be a predictor. The sudden shift to exclusive online instruction and learning methods rendered the students dissatisfied with their learning experience. The main reasons that might have influenced this dissatisfaction with online learning were technological difficulties, lack of internet access, connection problems, lost data and outage schedule, which can result in students being unable to even catch the classes or their exams online, and also technological ineptitude. 

An interesting qualitative study analyzed South Korean college students’ experiences of emergency remote teaching as a result of COVID-19, using thematic analysis [28]. The main reasons for perceiving a high degree of satisfaction with emergency remote teaching were, among others, the ability to access a place that facilitates concentration, with enough space, limiting mobility within the campus, saving time when traveling, classes first thing in the morning as well as good communication without interference. On the contrary, the main areas of dissatisfaction with emergency remote learning were the following: connection problems, delay in the arrival of the signal, asynchronization between the teacher’s voice and the teaching materials, lack of interaction, difficulty in communicating, difficulty maintaining attention due to classes for long periods of time and restrictions on practice or experiments.

Other authors recommend some suggestions to higher education establishments in order to increase the satisfaction of the e-learning environment with said accessibility of the e-learning portal 24/7, error-free information, quality of information and content, robustness of the server, training module materials related to the use of the e-learning portal for new users, updated information, well organized data, user-friendly portal design and occasional user comments [33].

With regard to the experience of the situation as beneficial reported in our study, a protective factor can be explained from the framework of resilience theory that acts as a buffer against the effects of traumatic experience, which enhances individual adaptation and positively influences successful adaptation and coping [34]. In fact, resilience, positive thinking, and exercise were identified as important coping strategies that predicted less severe mental health impacts in the study by Lai et al. [28]. Psychological rumination about the pandemic and resilience has significant positive and negative direct effects on depressive symptoms, respectively, where specific interventions maybe needed to address several factors in order to observe major improvements in one’s mental health outcomes. For example, cognitive reappraisal can mitigate the onset of fatigue and depressive symptoms [35].

As in our study, the economic impact has also been observed in another studies, as in the case of Kapasia et al. [36], in which 181 out of 232 students reported that their economic condition would be affected by the COVID-19 pandemic and 178 students reported that low family income amidst COVID-19 would have a negative impact on their education and may cause them to discontinue education.

In terms of sociodemographic variables, a research team from India explored satisfaction levels among 1182 students, in the course of which an in-depth analysis showed that satisfaction levels varied significantly according to different age groups. There were 51.6% negative online class reviews from subjects in the ‘18–22’ age group, compared to 31.5% negative reviews from subjects in the ‘7–17’ age group who spent more time in online classes [37].

### 4.2. Staff

Most of the university staff experienced an increase or the same level of stress as before lockdown. With regard to quality of life, half of the sample reported that it remained the same, although, as in the case with students, feelings of depression and anxiety increased throughout the weeks of lockdown.

After regression analysis, professional experience was explained by country of residence, social life, stress week 1–2, depression/anxiety week > and experience considered beneficial. In accordance with machine learning analysis, the relevant variables were as follows: quality of life week >5, experience considered beneficial, whether exams were postponed or cancelled, childcare and type of accommodation. Online teaching was predicted by: social life, stress week 1–2, teaching continuity, financial difficulties and loss of employment (regression analysis), and by financial difficulties, full/part time job, depression/anxiety week >5, anxiety about funding of research projects, coexistence problems, depression/anxiety week 3–4, quality of life week 1–2, lost loss job and country of residence.

The rapidly changing work and study arrangements were deemed to cause work-related stress, which might be aggravated by personal stressors such as having to work remotely, having to change tasks and having to combine all of this with home schooling children and caring for elderly family members [38]. 

Very few studies have analyzed the effect of lockdown on university staff. The Spanish study [4] on 586 administrative staff faculty members and academic staff showed that they evidenced significant lower anxiety and depression levels compared to students. In contrast, university employees from all groups obtained scores of greater concern regarding their own health, partner’s health, parents’ health, children and friends’ health and their social and economic situation, except for concern about relatives’ health. Along the same lines, a study carried out in Italy show that lockdown had a significant impact on psycho-emotional well-being and was greater in students than in administrative staff workers, and in females than in males [39].

Another study analyzed the emotional impact of COVID-19 via an online survey in one thousand and fifty-five staff and observed that 22–24% of them reported clinical-level anxiety and depression levels. Staff experienced high stress levels due to COVID-19 (66.2%, labeled vulnerable) and 33.8% experienced low stress levels (labeled resilient). Predictors of vulnerability in staff were the fact of having children and social isolation, while resilience was predicted by exercise in staff [38].

In our study, the chi-square automatic interaction detector analysis showed that the professional experience is either positive or staff does not suffer any impact in the groups of participants, with an increase in or maintaining of quality of life from week 5, perception of benefits derived from lockdown, as well as access to services and products. Resilience was seen as the ability to overcome adversity, which can be shown as experiencing no impact or positive impact on stress levels due to COVID-19 and by functioning well at work. It may be that for some, the crisis brought some benefits as well, for example, no longer having to travel or being able to work from a relatively quiet workplace at home [38]. 

The strength of this study lies in its target group, namely 1084 university students and 554 staff in total from four different countries (Spain, Colombia, Chile and Nicaragua), affiliated with nine different universities, four of them Spanish and one of which was online. On a methodological level, the study uses classic statistical analysis techniques and is one of the first to provide analyses based on artificial intelligence and machine learning that focuses on an analysis of variables that predict educational experience and online teaching. Another of our study’s strengths is that it takes into account the time factor in terms of the evolution of the perception of stress, anxiety, depression and quality of life during lockdown. In other words, it provides longitudinal data about one’s emotional state during lockdown by controlling the influence of different phases and regulations regarding confinement that affect the different countries involved in the study. Additionally, although there have been a range of studies about the experiences of university students, there has been little research into university staff in particular.

### 4.3. Limitations and Future Lines 

Regarding limitations, the first is linked to the quantity and quality of data, as this cannot be representative of the total sample of Spanish-speaking university students and staff. Furthermore, the questionnaires are common to them, and the disparity in terms of phases and regulations in each country in terms of the evolution of the pandemic provides evidence of possible bias when interpreting the results. 

From the standpoint of technological limitations of the tools used to analyze data, we should like to draw attention to a question that emerges. It proved necessary to use a linear kernel in the support vector machine algorithm in order to find the respective coefficients, with a view to detecting any trends in the response variables with regard to input attributes. In this respect, the results obtained are limited insofar as a linear relationship exists between factors. 

By way of conclusion, the combination of traditional statistical techniques and machine learning enable the key elements to be validated within the experiences of university students and staff during the lockdown process in terms of their educational/professional experience and online learning teaching.

## 5. Conclusions

The study has shown evidence of the effects of the pandemic on various aspects of the quality of life of university students and staff, specifically in the context of Latin American countries. Regardless of the fact that the effects of the pandemic can be specifically expressed in the contexts of countries, and even in regions within countries, the results show that the characteristics of the populations related to universities determine that they have broadly been affected in areas related to learning, work performance and quality of life. The fact that the pandemic has conditioned the mass use of information technologies almost exclusively should influence the exploration of alternatives to help improve their implementation on a wider scale, but also help us consider more appropriate options to minimize the adverse reactions that have been triggered.

Given that the results of this study were obtained in the first phase of the pandemic, it would be of special significance to research the evolution of the effects described, and these variations should especially be analyzed in countries where the pandemic has not been controlled at any time, as is the case of the Latin American countries included in this study (Colombia and Chile). 

From a public policy perspective, the study highlights two aspects of particular relevance. First, the priority that the evaluation of the impact of the pandemic on the living conditions of the people who are related to them, as well as on the practices developed to implement control measures, should be a priority for those responsible for the management of universities. Second, given that the effects of the pandemic could last for varying periods of time, it is especially important to establish procedures for monitoring the effects on both students and university staff.

## Figures and Tables

**Table 1:** Student sample description.

Sample Size n = 1084	n	%
**Sex** Male Female	698 358	65% 33%
**Age (years)**	24.1	DT (7.7)
**Type of university study** Undergraduate Graduate	899 185	83% 17%
Face to face Online	899 185	83% 17%
**Country** Spain Colombia Chile Nicaragua	558 440 64 22	51.5% 40.6% 6% 2%
**My educational experience** Negatively impacted Not impacted Positively impacted NA	713 228 79 64	66% 21% 7.3% 6%
**Online learning experience** Online learning is possible but in person it is better Online learning was not a good experience Online learning is great and should continue	545 325 102	50.3% 30% 9.4%
**My university progressed with the exams** Yes No NA	877 93 114	81% 8.6% 10.5%
**After the university closed** I continued learning online Continuing learning online was not possible NA	925 66 93	85% 6.1% 8%
**University was supportive** Yes No NA	705 314 65	65% 29% 6%
**Social life** Social life suffered but I had support Negatively impacted Positively impacted NA	593 266 177 48	55% 24.5% 16.3% 4.4%
**Financial difficulties** No Yes NA	520 500 64	48% 46% 6%
**Coexistence problems at home** No Yes NA	544 458 82	50% 42% 7%
**Chronic disease** No Yes	931 134	86% 12%

Note: NA = no answer/not applicable.

**Table 2:** Staff sample description.

Sample Size n = 554	n	%
**Sex** Male Female	193 334	35% 60%
**Age (Years)**	32.6	DT (13.7)
**Type of university** Face to face Online NA	504 29 21	91% 5% 4%
**Country** Spain Colombia Chile Nicaragua	376 125 10 43	68% 23% 2% 8%
**Size of city/town** Countryside/suburb Large city Small city/town	41 319 177	7.4% 58% 32%
**Lockdown was beneficial** No Yes NA	329 134 91	59.4% 24.2% 16.4%
**My professional experience** Negatively impacted Not impacted Positively impacted NA	204 196 128 26	37% 35.4% 23% 4.7%
**Online teaching experience** Online teaching is possible but in person it is better Online teaching was not a good experience Online teaching is great and should continue NA	279 127 78 70	50.5% 23% 14% 12%
**Teaching continuation** I continued teaching online It was not possible NA	423 8 97	76.4% 1.5% 17%
**Social life** Negatively impacted Social life suffered but I had support Positively impacted	110 305 105	20% 55% 19%
**Financial difficulties** No Yes NA	297 182 75	54% 33% 13.5%

Note: NA = no answer/not applicable.

**Table 3:** Regression analysis for students.

	Educational Experience			Online Learning		
	B	SE	β	B	SE	β
(Constant) Gender Age Country of Residence Size of town/city Month University Presence Online Chronic Disease I am a special needs student Family Resources Social Life Couple Relationship Coexistence Problems Psychological/Physical Abuse Stress Week 1–2 Stress Week 3–4 Stress Week >5 Quality of Life Week 1–2 Quality of Life Week 3–4 Quality of Life Week >5 Depression/Anxiety Week 1–2 Depression/Anxiety Week 3–4 Depression/Anxiety Week >5 Beneficial Physical Activity Teaching continuation University was supportive University progressed with exams Exams were postponed Financial difficulties Access to Products and Services	1.408 −0.062 0.008 −0.005 −0.012 −0.002 −0.059 −0.018 0.002 0.016 0.134 0.035 −0.049 0.203 0.040 −0.043 0.000 −0.035 0.084 −0.015 0.019 −0.047 −0.121 0.055 −0.010 −0.085 −0.122 −0.063 0.030 −0.109 0.109	0.333 0.039 0.002 0.003 0.028 0.025 0.064 0.054 0.078 0.019 0.031 0.015 0.039 0.079 0.027 0.029 0.027 0.032 0.036 0.031 0.040 0.043 0.041 0.032 0.013 0.036 0.041 0.031 0.030 0.039 0.039	−0.048 0.096 * −0.062 −0.012 −0.002 −0.027 −0.010 0.001 0.025 0.140 ** 0.067 * −0.039 0.077 * 0.048 −0.046 0.000 −0.039 0.095 * −0.018 0.016 * −0.038 −0.096 * 0.051 −0.024 −0.070 * −0.090 * −0.061 * 0.030 * −0.087 * 0.088 *	1.503 0.030 0.026 0.001 0.016 0.029 0.014 −0.094 −0.059 −0.007 0.045 −0.023 −0.032 0.010 0.050 −0.043 0.013 0.045 0.025 0.016 0.008 −0.026 −0.121 0.042 0.000 −0.116 −0.219 −0.032 0.080 −0.023 0.030	0.361 0.043 0.003 0.003 0.031 0.027 0.069 0.059 0.085 0.021 0.033 0.017 0.043 0.086 0.029 0.031 0.029 0.035 0.039 0.034 0.043 0.046 0.045 0.035 0.014 0.039 0.045 0.034 0.033 0.042 0.042	0.021 0.289 ** 0.012 0.014 0.031 0.006 −0.046 −0.021 −0.010 0.043 −0.039 −0.023 0.003 0.055 −0.041 0.014 0.045 0.025 0.017 0.006 −0.019 −0.087 0.034* −0.001 −0.086 * −0.146 ** −0.028 0.071 * −0.017 0.022
R^2^ Durbin–Watson VIF	0.16 2.07 <1.6			0.20 1.9 <1.6		

Note: * *p* < 0.05; ** *p* < 0.001.

**Table 4:** Regression analysis for staff.

	Professional Experience Was			Online Teaching Was		
	B	SE	β	B	SE	β
(Constant) Age Gender Month Country of Residence Size of town/city Family Resource Chronic Disease Social Life Couple Relationship Coexistence Problems Access to Services and Products Physical Activity Stress Week 1–2 Stress Week 3–4 Stress Week >5 Quality of Life Week 1–2 Quality of Life Week 3–4 Quality of Life Week >5 Depression/Anxiety Week 1–2 Depression/Anxiety Week 3–4 Depression/Anxiety Week >5 Beneficial Teaching Continuation My University was supportive Financial difficulties I lost my job Psychological/Physical Abuse Childcare significantly impacted my work	2.661 0.001 −0.069 −0.011 −0.020 −0.074 0.031 0.040 0.185 0.022 −0.084 0.079 −0.012 0.114 −0.087 0.017 −0.021 −0.104 0.037 −0.094 −0.022 −0.177 0.163 0.002 −0.108 −0.082 −0.087 −0.195 −0.041	0.620 0.003 0.065 0.047 0.005 0.058 0.037 0.081 0.059 0.034 0.095 0.070 0.024 0.049 0.048 0.043 0.060 0.073 0.060 0.073 0.077 0.074 0.051 0.072 0.099 0.112 0.079 0.292 0.067	0.019 −0.044 −0.010 −0.166 ** −0.053 0.035 0.020 0.144 * 0.027 −0.039 0.049 −0.021 0.120 * −0.090 0.018 −0.019 −0.100 0.037 −0.063 −0.015 −0.120 * 0.135 * 0.001 −0.046 −0.035 −0.050 −0.029 −0.025	2.145 −0.003 −0.111 −0.030 0.008 −0.030 0.032 0.016 0.119 −0.040 0.128 −0.030 0.008 0.099 −0.083 0.037 −0.010 −0.060 0.081 −0.064 −0.025 −0.070 0.010 0.639 −0.010 −0.328 0.142 −0.209 0.091	0.576 0.003 0.060 0.044 0.005 0.054 0.034 0.075 0.055 0.031 0.089 0.065 0.022 0.045 0.045 0.040 0.056 0.068 0.056 0.068 0.071 0.068 0.048 0.067 0.092 0.104 0.073 0.271 0.062	−0.045 −0.074 −0.027 0.067 −0.022 0.037 0.009 0.095 * −0.050 0.062 −0.019 0.014 0.108 * −0.089 0.042 −0.009 −0.060 0.084 −0.044 −0.017 −0.049 0.009 0.378 ** −0.004 −0.146 * 0.085 * −0.032 0.058
R2 Durbin–Watson VIF	0.16 1.9 <1.6			0.23 2.03 <1.6		

Note: * *p* < 0.05; ** *p* < 0.001.

**Table 5:** Reduction in Dimensionality.

Interval	Correlated Variable 1	Correlated Variable a 2	Variable Selected
0.5	Are you still considering going abroad if accepted?	Was your plan to study abroad?	Are you still considering going abroad if accepted?
|0.4| && rho < |0.5|	I am anxious about my job security	I lost my part time employment due to the pandemic	I am anxious about my job security
	I am anxious about my job security	Financial difficulties	I am anxious about my job security

**Table 6:** Educational Experience of Students.

Variables Selected
Stress Week >5
Beneficial
I am experiencing financial difficulties due to the pandemic
Depression Anxiety Week >5
My university was supportive in offering services which enabled me to continue
Stress Week 1–2
Social Life
Quality of Life Week 3–4

**Table 7:** Learning Experience of Students.

Variables Selected
Stress Week >5
Beneficial
Quality of Life Week 3–4
My university was supportive in offering to continue services

**Table 8:** Professional experience.

Variables Selected
Beneficial
Quality of Life Week < 5
Exams were postponed or cancelled
Academic_Non-academic
Childcare significantly impacted my education work
Accommodation

**Table 9:** Online Teaching Experience of Staff.

Variables Selected
Financial Difficulties
Full/Part Time Job
Depression/Anxiety Week >5
Anxiety about funding of my research projects
Coexistence problems
Depression/Anxiety Week 3–4
Quality of Life Week 1–2
I lost my job
Country

**Table 10:** Educational/Professional Experience of Students + Staff.

Variables Selected
Student_Staff
I was prior to the pandemic employed
Depression_Anxiety Week 5+
Child care significantly impacted my education work
Academic_Non-academic

**Table 11:** Online Learning/Teaching Experience of Students + Staff.

Variables Selected
My university progressed with exams and relevant changes were made
My university was supportive in offering to continue services
Academic_Non-academic
I am graduating and actively applying for jobs
After the University ClosedTeaching Learning

## Data Availability

Data were obtained from Osipenko [16].
